# Characteristics of Pos19 – A Small Coding RNA in the Oxidative Stress Response of *Rhodobacter sphaeroides*

**DOI:** 10.1371/journal.pone.0163425

**Published:** 2016-09-26

**Authors:** Katrin M. H. Müller, Bork A. Berghoff, Benjamin D. Eisenhardt, Bernhard Remes, Gabriele Klug

**Affiliations:** Institut für Mikrobiologie und Molekularbiologie, Justus-Liebig-Universität, Heinrich-Buff-Ring 26, 35392, Giessen, Germany; Universite Paris Descartes, FRANCE

## Abstract

The phototrophic bacterium *Rhodobacter sphaeroides* induces several small RNAs (sRNAs) when singlet oxygen (^1^O_2_) levels are elevated, a situation also referred to as photo-oxidative stress. An RNA-seq study identified the RSs0019 sRNA, which is renamed Pos19 (photo-oxidative stress induced sRNA 19). Pos19 is part of the RpoE regulon and consequently induced upon ^1^O_2_ and peroxide stress. The 219 nt long Pos19 transcript contains a small open reading frame (sORF) of 150 nt, which is translated *in vivo*. Over-expression of Pos19 results in reduced mRNA levels for several genes, of which numerous are involved in sulfur metabolism. The negative effect on the potential targets is maintained even when translation of the sORF is abolished, arguing that regulation is entailed by the sRNA itself. Reporter studies further revealed that regulation of the most affected mRNA, namely RSP_0557, by Pos19 is Hfq-dependent. Direct binding of Pos19 to Hfq was shown by co-immunoprecipitation. Physiological experiments indicated Pos19 to be involved in the balance of glutathione biosynthesis. Moreover, a lack of Pos19 leads to elevated reactive oxygen species levels. Taken together our data identify the sRNA Pos19 as a coding sRNA with a distinct expression pattern and potential role under oxidative stress in the phototrophic bacterium *R*. *sphaeroides*.

## Introduction

The accumulation of molecular oxygen in the earth’s atmosphere at around 2.4–2.3 billion years ago changed the fate of biological evolution dramatically. Organisms that developed under anaerobic conditions suddenly had to cope with oxidative stress caused by reactive oxygen species (ROS). Those organisms which survived the "Great Oxidation Event" developed protection mechanisms to handle this specific threat. Among the ROS produced throughout metabolism, singlet oxygen (^1^O_2_) is unique because its formation does not rely on electron but rather energy transfer to molecular oxygen. Since light is pivotal for this energy transfer, ^1^O_2_ stress is often referred to as photo-oxidative stress. Another prerequisite for its generation is the existence of a photosensitizer that is capable of transferring light energy to molecular oxygen. In phototrophic organisms naturally occurring photosensitizers are chlorophyll and bacteriochlorophyll (BChl) molecules as well as their porphyrin precursors (for review on ^1^O_2_ generation see [1]). In the anoxygenic, phototrophic bacterium *Rhodobacter sphaeroides* triplet state BChl *a* molecules of the reaction centre generate ^1^O_2_ in perceivable amounts [2], while carotenoids carry out a protective function by quenching either excited BChl *a* or ^1^O_2_ directly [3,4]. In addition to the existence of carotenoids in photosynthetic membranes, *R*. *sphaeroides* harbors a complex regulatory network to manage the photo-oxidative stress response and to finally adapt to ^1^O_2_. The photo-oxidative stress response is initiated by the release of the extracytoplasmic function sigma factor RpoE from its inhibitory anti-sigma factor ChrR which is degraded upon stress [5,6,7]. The set of genes that are directly targeted by RpoE is rather small (~15 genes) [8] but also includes another alternative sigma factor gene, *rpoH*_*II*_ [9,10]. The RpoH_II_ regulon itself may comprise up to 100 genes and additionally shares an overlapping regulon with the heat shock sigma factor RpoH_I_ [11]. Genes within the RpoH_II_ regulon provide functions in glutathione (GSH) dependent defense against ^1^O_2_, detoxification of methylglyoxal and ^1^O_2_ quenching [12]. When present in elevated amounts, ^1^O_2_ is highly toxic and causes severe damages of macromolecules, albeit proteins are the primary targets. Products of the so called indirect photo-oxidation of proteins comprise tryptophan, tyrosine and histidine peroxides as well as methionine sulfoxides and disulfides formed from cysteines [13]. In subsequent reactions toxic hydroperoxides in proteins and lipids can evolve as secondary damages. A further source for oxidative stress in photosynthetic organisms is iron limitation which strongly increases the intracellular ROS levels [14,15]. These increased ROS levels occur since iron is essential for enzymes involved in ROS detoxification such as catalases and Fe-containing superoxide dismutase and for oxidative stress sensing enzymes such as SoxRS. Therefore *R*. *sphaeroides* and other photosynthetic organisms have a need for sophisticated response and protection mechanisms in order to survive oxidative stress.

In former studies we have shown that *R*. *sphaeroides* expresses several sRNAs upon ^1^O_2_ stress, which are under control of RpoE, RpoH_II_ and/or RpoH_I_ [16,11]. So far the function in stress responses of sRNAs in *R*. *sphaeroides* could be verified for at least three examples: the sRNA SorY (former RSs1543), which by limiting the malate import contributes to the balance of metabolic fluxes [17], and the sRNAs CcsR1-4 (former RSs0680a-0680d), which affect C1 metabolism upon oxidative stress conditions [18]. Recently, we described the 3’UTR-derived sRNA SorX (former RSs2461), which counteracts oxidative stress by regulating spermidine uptake [19]. From other bacterial models it is known that sRNAs have a central role in stress regulatory networks by modulating mRNA stability and translation [20,21]. An impressive example is the modulation of the outer membrane by manifold sRNAs in enterobacteria [22]. The RNA chaperone Hfq regularly supports functionality of sRNAs by facilitating sRNA-mRNA interactions and by impairing turnover of transcripts [23,24]. Since Hfq interacts with plenty of sRNAs, it represents a global regulator. In *R*. *sphaeroides* Hfq affects several cellular processes, amongst others the photo-oxidative stress response [25]. The three RpoH_I_/RpoH_II_-dependent sRNAs (SorY, CcsR1, and SorX) could be co-immunoprecipitated with Hfq and consequently might have caused the higher ^1^O_2_ sensitivity of the *R*. *sphaeroides hfq* deletion mutant [25].

In this study, we describe the diverse characteristics of the sRNA RSs0019 and rename it as Pos19 (photo-oxidative stress induced sRNA 19) for its primary noted appearance upon photo-oxidative stress. The sRNA is furthermore induced by peroxide stress and iron limitation. The small open reading frame (sORF) within the Pos19 sequence was shown to be translated *in vivo*. Pos19 over-expression combined with microarray analysis identified Pos19-regulated genes and mutational analyses further revealed that regulation does not depend on translation of the sORF. Albeit the interaction with Hfq appears to be rather weak, Pos19 can be co-immunoprecipitated with Hfq and relies on Hfq for regulation of RSP_0557, encoding a hypothetical protein. Regarding a Pos19 function in oxidative stress responses, it was demonstrated that it alters the intracellular GSH level most likely by affecting the mRNA level of several sulfur metabolism genes. Moreover, a lack of Pos19 resulted in increased cellular ROS levels and caused a slight growth defect under iron limitation, a condition also leading to increased ROS levels. These findings lead to the assumption that Pos19 is involved in a complex regulatory circuit needed to balance metabolic processes such as GSH biosynthesis and ROS detoxification upon oxidative stress. Furthermore, our data suggest Pos19 to be a regulatory sRNA with a coding function.

## Results

### Pos19 is an RpoE- and oxygen-dependent sRNA with a coding function

**Fig 1:**
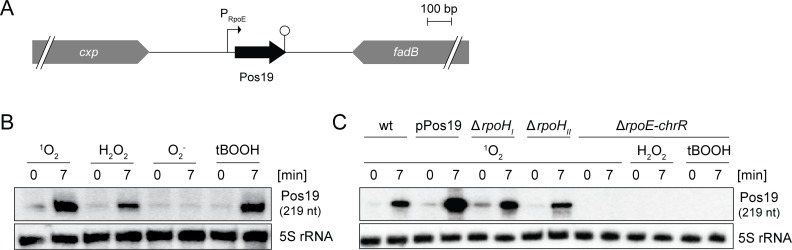
Pos19 is induced by singlet oxygen and peroxides. (A) Graphical representation of the Pos19 locus in *R*. *sphaeroides* wild-type strain 2.4.1. The *pos19* gene is located on chromosome 2 between *cxp* and *fadB*. The conserved RpoE-dependent promoter (P_RpoE_) and the Rho-independent terminator (lollipop structure) are indicated. (B) Northern blot for stress-dependent Pos19 induction. *R*. *sphaeroides* wild-type 2.4.1 cultures were treated with stress-generating chemicals and samples collected at time points 0 and 7 min. Singlet oxygen (^1^O_2_) was generated by the addition of 0.2 μM methylene blue in the presence of high light intensities (800 W m^-2^). Hydrogen peroxide (H_2_O_2_) and *tert*-butyl hydroperoxide (tBOOH) were added in final concentrations of 1 mM and 300 μM, respectively. 250 μM of paraquat (PQ) were used for the generation of superoxide radicals (O_2_^‒^). 5S rRNA was probed as loading control. (C) Northern blot for RpoE-dependent Pos19 expression. The *R*. *sphaeroides* wild-type (wt), the Pos19 over-expression (pPos19), RpoH_I_ and RpoH_II_ mutant strains, as well as a strain lacking the *rpoE-chrR* locus (TF18), were treated with the indicated stress-generating chemicals as described above. Samples were collected at the indicated time points. 5S rRNA was probed as loading control.

**Fig 2:**
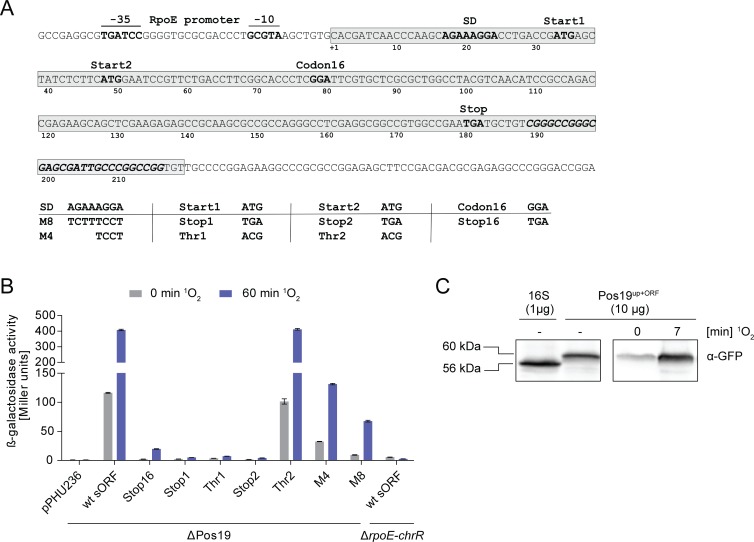
Pos19 is a coding sRNA. (A) Detailed illustration of the *pos19* gene. Relevant features are shown in bold. The -35 and -10 motifs of the RpoE-dependent promoter are marked. The 219 nt long sRNA (shaded in grey) contains two potential ORFs with lengths of 150 (Start1) and 135 nt (Start2). The Rho-independent terminator (italic letters) was predicted by TransTermHP [27] and correlates with the 3’ end mapped by 3’ RACE. The Shine-Dalgarno (SD) sequence, the two start codons as well as one internal codon (Codon16) were mutated according to the chart below the sequence. (B) *In vivo* translation of the Pos19-sORF was monitored by translational *lacZ* fusions. A 300 bp fragment containing the RpoE-dependent promoter and the first 178 bp of the *pos19* gene (including codons 1–48) was fused to the promoter-less *lacZ* gene on reporter plasmid pPHU236. The wild-type (wt) sORF was subsequently mutated as shown in (A). The fusion plasmids and control plasmid pPHU236 were transferred to *R*. *sphaeroides* ΔPos19 or strain TF18, which lacks the *rpoE-chrR* locus. The corresponding strains were subjected to ^1^O_2_ stress for 60 min with samples collected at the indicated time points. ß-galactosidase activities were measured from biological triplicates with technical duplicates. Error bars represent the standard error of the mean. (C) *In vivo* translation of the Pos19-sORF was monitored by translational eCFP fusion. The Pos19 peptide, C-terminally fused to eCFP (Pos19^up+ORF^), was detected with a polyclonal anti-GFP antibody on a Western blot. Cultures were stressed as described for Fig 1 and samples were withdrawn at the indicated time points. The amount of total protein extracts is shown in brackets. Protein from a strain carrying the pBE_eCFP::eCFP with 16S rRNA promoter (16S) was used as control. The size difference between control and Pos19 fusion of around 4 kDa corresponds to molecular weight of the predicted Pos19 peptide.

Pos19 was first identified as RSs0019 in a global screen for sRNAs in *R*. *sphaeroides* by differential RNA-sequencing (dRNA-seq) [16]. The primary 5’ end of Pos19 was unambiguously determined by the dRNA-seq approach. In addition, we mapped the 3’ end by 3’ RACE, which validated a size of 219 nt. The detected 3’ end correlates with a predicted Rho-independent terminator (Figs 1A and 2A). Interestingly, the terminating structure misses the typical 3’ polyU stretch that is found in many bacterial sRNAs. It was already demonstrated that ^1^O_2_, but not superoxide radicals (O_2_^‒^), induce Pos19 and that the alternative sigma factor RpoE is responsible for this induction [16]. Pos19 is preceded by a perfectly conserved RpoE-dependent promoter (TGATCC(N_15_)GCGTA; Figs 1A and 2A), which can be targeted by RpoE when the inhibitory interaction with its cognate anti-sigma factor ChrR is released upon ^1^O_2_ stress. Additional stress factors lead to RpoE activation: peroxides like hydrogen peroxide (H_2_O_2_) and organic hydroperoxides act as signals for RpoE activation [26,7]. In line with these observations, Pos19 is clearly induced after treatment with H_2_O_2_ and the organic hydroperoxide tBOOH (*tert*-butyl hydroperoxide) in an RpoE-dependent manner but independently of RpoH_I_ and RpoH_II_ (Fig 1B and 1C). In strain TF18, lacking the *rpoE-chrR* locus, there is no induction of Pos19 under the tested stress conditions, while in the RpoH_I_ and RpoH_II_ mutant strains the expression of Pos19 is not altered upon ^1^O_2_ stress compared to the wild-type.

When inspecting the Pos19 sequence, we found two putative sORFs with lengths of 135 and 150 nt, depending on the particular start codon (Start1 and Start2; Fig 2A). To address the question whether translation occurs *in vivo*, a translational fusion to *lacZ* on reporter plasmid pPHU236 was constructed and subsequently mutated (Fig 2A). The wild-type fusion gave a β-galactosidase activity of 116 Miller units (MU) before stress, which was increased to 408 MU after 60 min of ^1^O_2_ stress. In strain TF18, which lacks the *rpoE-chrR* locus, reporter activity of the wild-type fusion was negligible (Fig 2B). As negative control, we used a strain carrying the control plasmid pPHU236 with the promoter-less *lacZ* gene, which completely lacked activity. Mutations in the first start codon (Stop1 and Thr1) as well as mutations that introduce an internal stop codon (Stop2 and Stop16) nearly abolished β-galactosidase activity. In contrast, mutating the second start codon to ACG (Thr2) did not affect reporter activity. These findings rather argue for the first start codon to be essential for translation initiation. A potential Shine-Dalgarno (SD) sequence was found shortly upstream of the first start codon, however, SD mutations (M4 and M8) did not completely abolish translation and reporter activities remained at fairly high levels especially under stress conditions (Fig 2B). In summary, translation initiation of the Pos19-sORF occurs at the first start codon and partly relies on an intact SD sequence. To verify the *in vivo* translation, a translational fusion of the Pos19-sORF, carrying its own promoter, to eCFP on the reporter plasmid pBE_eCFP::eCFP was constructed (pBE_Pos19^up+ORF^::eCFP). Western blot analysis verified the expression of the Pos19 peptide upon ^1^O_2_ stress and validated the β-galactosidase activity assay (Fig 2C). The Pos19 sequence with SD and sORF is conserved in different *R*. *sphaeroides* strains (S1 Fig) but was not found in other species.

### The Pos19 regulon comprises genes involved in sulfur metabolism

**Fig 3:**
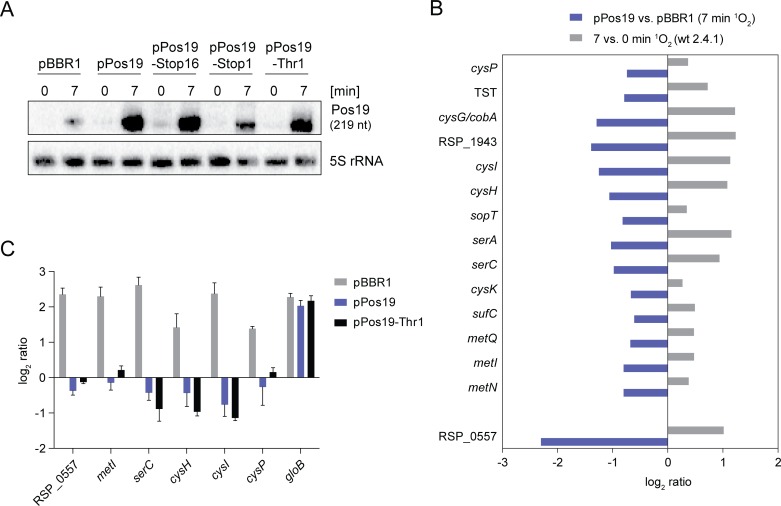
Genes involved in sulfur metabolism are repressed by Pos19 upon ^1^O_2_ stress. (A) Plasmid-borne over-expression of Pos19. The *pos19* gene together with its native RpoE-dependent promoter was cloned into middle-copy plasmid pBBR1 and expressed in *R*. *sphaeroides* wild-type 2.4.1 cells (pPos19). Additionally, Pos19 was over-expressed with mutations in codon 16 and start codon 1 (Fig 2A). Wild-type cells carrying empty vector pBBR1 served as a control (pBBR1). Strains were subjected to ^1^O_2_ stress experiments and samples (collected at 0 and 7 min) used for Northern blot analysis. The 5S rRNA was probed as a loading control. (B) Microarray results for genes involved in sulfur metabolism and the most down-regulated mRNA RSP_0557. Samples from the Pos19 over-expression strain (pPos19) were collected after 7 min of ^1^O_2_ stress and compared to an empty vector control (pBBR1) by microarray analysis (blue bars). In a second microarray analysis, samples from stressed *R*. *sphaeroides* wild-type 2.4.1 cells (7 min ^1^O_2_) were compared to unstressed cells (grey bars) [28]. Effects on mRNA levels were calculated as log_2_ ratios. Values represent the mean from two individual microarray analyses, each containing pooled biological triplicates per strain. (C) Induction of selected genes by qRT-PCR. Empty vector control (pBBR1) and over-expression strains (pPos19 and pThr1) were subjected to ^1^O_2_ stress experiments with samples collected at time points 0 and 7 min of stress. Selected mRNA levels in stress samples (7 min) were calculated relative to unstressed samples (0 min) as log_2_ ratios, using *rpoZ* as a control gene. Results were obtained from three independent biological experiments. Error bars represent the standard error of mean.

To learn more about the function of Pos19, we constructed an over-expression strain. For this purpose, the *pos19* gene together with its native RpoE-dependent promoter was inserted into the middle-copy plasmid pBBR1 (pPos19) and expressed in the *R*. *sphaeroides* wild-type background. The corresponding strain consequently exhibits a ^1^O_2_-induced Pos19 over-expression compared to an empty vector control strain (Fig 3A). Total RNA from the over-expression (pPos19) and empty vector control (pBBR1) strain was extracted after 7 min of ^1^O_2_ stress and used for microarray analysis to investigate Pos19-induced changes on transcriptome level. All mRNAs which showed an up- or down-regulation with a log_2_ ratio ≥ 1.0 or ≤ -1.0 are shown in Table 1. Among the up-regulated mRNAs we found *fliC* (flagellar filament protein), the *cox* operon (RSP_2876–79) and three genes for hypothetical proteins (RSP_2590, RSP_3469, and RSP_6085). Among the down-regulated mRNAs, RSP_0557 (encoding a hypothetical protein) was most strongly affected by Pos19 over-expression (Fig 3B). In addition, mRNAs from genes/operons with a function in serine (*serC*, *serA*) and sulfur metabolism (*cysH*, *cysI*, RSP_1943, *cysG/cobA*) showed decreased mRNA levels. Since serine can easily enter cysteine metabolism pathways we carefully re-inspected the microarray data for down-regulated mRNAs (log_2_ ratio ≤ -0.6) with a function in sulfur or cysteine metabolism (Fig 3B). In total, 30 genes were down-regulated with a log_2_ ratio ≤ -0.6, 14 of which had a direct or indirect function in sulfur metabolism (S1 Table). Besides the above-listed mRNAs from the *cys* operon (RSP_1944–39), which is involved in assimilatory sulfate reduction, we found *cysP* from the *cysAPTW* operon (RSP_3696–99), which encodes an ABC sulfate/thiosulfate transporter, to be down-regulated. Moreover, *cysK* (cysteine synthase), *sopT* (sulfate adenylyltransferase) and TST (Rhodanese-related sulfur transferase) were decreased in mRNA level (see S2 Fig for their particular functions in sulfur metabolism). Finally, the *metNIQ* operon, encoding an ABC D-methionine transporter, was down-regulated. This is of interest because methionine is a sulfur-containing amino acid. The microarray data for Pos19-affected mRNAs involved in sulfur metabolism and the most affected gene RSP_0557 were compared to microarray ratios calculated from experiments with *R*. *sphaeroides* wild-type 2.4.1 cultures before (0 min) and after 7 min of ^1^O_2_ stress (Fig 3B) [28]. Intriguingly, the mRNAs which are repressed upon Pos19 over-expression are slightly or clearly up-regulated under ^1^O_2_ stress in the wild-type background (Fig 3B). To exclude the possibility that changes observed in the Pos19 microarray were solely due to the action of the small peptide translated from the Pos19 transcript, we constructed over-expression strains for Pos19 containing sORF mutations that abrogate translation (Stop16, Stop1, Thr1; Fig 2A and 2B). Like pPos19, the pPos19 mutant constructs produced increased sRNA levels compared to the pBBR1 empty vector control (Fig 3A). We then monitored the induction of selected genes after 7 min of ^1^O_2_ stress in three strains by qRT-PCR (Fig 3C). The pBBR1 control strain revealed that RSP_0557 and five selected genes from sulfur metabolism (*metI*, *serC*, *cysH*, *cysI* and *cysP*) are induced upon ^1^O_2_ stress in *R*. *sphaeroides*. Since the control strain reflects the wild-type situation, this validated the microarray results shown in Fig 3B. When Pos19 was over-expressed the induction disappeared and mRNA levels remained unchanged or were even slightly reduced (Fig 3C). This was also the case when over-expressing Pos19 with the abolished sORF translation (Thr1; Fig 3C). As a control we monitored induction of *gloB* (RSP_0799), which is known to be induced upon ^1^O_2_ stress [9,12] and was not changed in the Pos19 microarray. As expected, induction was observed in all three strains (Fig 3C). From these experiments, we conclude that the effect of Pos19 on the selected genes is independent of the small peptide and displays a regulatory sRNA function. Surprisingly, mRNA levels of the selected genes were not further increased when deleting Pos19 and there was no significant difference compared to the wild-type (data not shown). Moreover, there was no effect when constitutively over-expressing Pos19 (pRK16S::Pos19) under non-stress conditions (data not shown).

**Table 1:** Genes with differential mRNA levels upon Pos19 over-expression, log_2_ ratios ≥ 1 and ≤ -1.0.

Gene Number	Gene Name	Log2 ratio pPos19 vs. pBBR1	Function
**Down-regulated**			
RSP_0557		-2.3	Hypothetical protein
RSP_1351	*serC*	-1.0	Phosphoserine aminotransferase
RSP_1352	*serA*	-1.0	D-3-phosphoglycerate dehydrogenase
RSP_1941	*cysH*	-1.1	Phosphoadenosine phosphosulfate reductase
RSP_1942	*cysI*	-1.3	Sulfite/nitrite reductase hemoprotein subunit
RSP_1943		-1.4	Sulfite reductase (ferredoxin)
RSP_1944	*cysG*/*cobA*	-1.3	Uroporphiryn-III C-methyltransferase/siroheme synthase
RSP_6037		-1.0	Hypothetical protein
**Up-regulated**			
RSP_0069	*fliC*	1.6	Flagellar filament protein
RSP_2590		1.2	Hypothetical protein
RSP_2876	*coxM*	1.2	Carbon monoxide dehydrogenase medium chain
RSP_2877	*coxL*	1.5	Carbon monoxide dehydrogenase large chain
RSP_2878	*coxS*	1.9	Carbon monoxide dehydrogenase small chain
RSP_2879	*coxG*	1.6	Carbon monoxide dehydrogenase subunit G
RSP_3469		1.1	Hypothetical protein
RSP_6085		1.0	Hypothetical protein

Log_2_ ratios were calculated from two individual microarray analyses, each containing pooled biological triplicates per strain. Reproducibility was high as reflected by a correlation coefficient (Pearson) of *r* = 0.92. Genes were considered to be differentially regulated when mRNA levels were changed with a log_2_ ratio ≥ 1.0 or ≤ -1.0 Genes located in an operon are grouped together.

### Pos19 affects cellular glutathione and ROS levels

**Fig 4:**
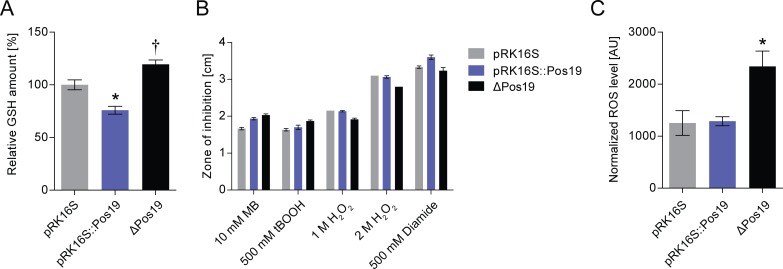
Pos19 influences GSH and ROS levels. (A) Measurement of total intracellular glutathione (GSH). GSH was measured using Ellmann’s reagent (DTNB) in cell samples from three *R*. *sphaeroides* strains: an empty vector control (pRK16S), a strain with a constitutive, plasmid-borne over-expression of Pos19 under the control of the 16S rRNA promoter (pRK16S::Pos19), and the Pos19 mutant (ΔPos19). The relative GSH amount is depicted in percent; the GSH amount of the empty vector control (pRK16S) was set to 100 percent. All results represent the mean of nine independent biological experiments with technical duplicates. Error bars reflect the standard error of mean. The level of significance compared to the control (pRK16S) is indicated († 0.05 < p < 0.1 and * p < 0.05). (B) Inhibition zone assay using 10 mM methylene blue (MB) in the light, 500 mM tBOOH, 1 M and 2 M hydrogen peroxide (H_2_O_2_), and 500 mM diamide_._ The diameter of the zones of inhibition was measured for the three *R*. *sphaeroides* strains described above for the GSH assay. The data represent the mean of three independent experiments with technical duplicates. The error bars represent the standard error of mean. (C) Effect of Pos19 on ROS levels. Determination of intracellular levels of ROS in the control strain (pRK16S) compared to Pos19 over-expression (pRK16S::Pos19) and mutant (ΔPos19). ROS generated by the cells were analyzed after reaction with 10 μM 2,7-dihydrodichlorofluorescein diacetate. The fluorescence intensity was normalized to the optical densities of the samples. The resulting values are presented in arbitrary units (AU). The data represent the mean of three independent experiments. The error bars indicate the standard error of mean. The level of significance compared to the control (pRK16S) is indicated (* p < 0.05).

Since sulfur metabolism genes were affected upon Pos19 over-expression, we took a closer look at the intracellular levels of the sulfur-containing antioxidant GSH. A Pos19 deletion mutant (ΔPos19), a strain constitutively over-expressing Pos19 (pRK16S::Pos19), and a strain carrying the respective empty vector (pRK16S) were compared. GSH assays revealed a significantly reduced amount of GSH in the Pos19 over-expression (76%) and an increased amount of GSH (119%) in the Pos19 deletion mutant (Fig 4A). Regarding these changes in the antioxidant GSH, we expected an effect on oxidative stress resistance in the corresponding strains. Surprisingly, we found only very minor differences in resistance that were not consistent with GSH measurements (Fig 4B). However, ROS levels were significantly increased in the Pos19 mutant while not changed in the over-expression strain (Fig 4C).

Transcriptome data from our group indicated that Pos19 is affected by iron concentrations. Further experiments revealed Pos19 to be up-regulated under iron limitation and demonstrated a slightly impaired growth of the Pos19 deletion mutant compared to the wild-type (S3 Fig).

### Pos19 is Hfq-dependent

**Fig 5:**
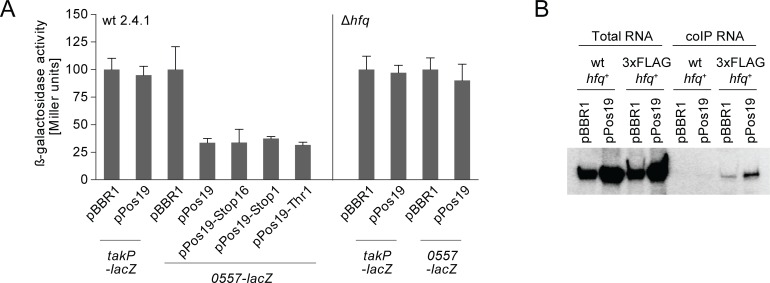
Regulation of RSP_0557 by Pos19 depends on Hfq. (A) Results from *in vivo* reporter studies. Gene fragments containing the first codons and part of the upstream region were translationally fused to the *lacZ* gene on plasmid pPHU235. The corresponding reporter plasmids were transferred to control (pBBR1) and over-expression strains of Pos19 (pPos19) in wild-type (wt 2.4.1) and *hfq* deletion mutant (Δ*hfq*) backgrounds. Cultures were stressed with ^1^O_2_ for 60 min and samples subjected to β-galactosidase assays. Bars indicate the relative β-galactosidase activity as calculated from Miller units (with the pBBR1 control set to 100% for each construct). Results represent the mean from three independent experiments with technical duplicates and error bars reflect the standard deviation. (B) Northern blot results from Hfq coIP experiments. Control plasmid pBBR1 and over-expression plasmid pPos19 were transferred to strains either expressing wild-type Hfq (wt *hfq*^+^) or 3xFLAG-tagged Hfq (3xFLAG *hfq*^+^). Resulting strains were exposed to ^1^O_2_ for 30 min at an OD_660_ of 0.4. Cell extracts were applied to total RNA (input) and coIP RNA (output) extraction. Six μg of total RNA and 350 ng of coIP RNA were loaded on polyacrylamide gels for detection of Pos19.

To further elucidate the regulatory potential of Pos19, we made use of an *in vivo* reporter system, in which mRNAs are translationally fused to the promoter-less *lacZ* gene on reporter plasmid pPHU235 and transcribed from a 16S rRNA promoter [23]. On a second plasmid, Pos19 is over-expressed from its native RpoE-dependent promoter (Fig 3A). A fusion of *takP* [17] was used as negative control and, as expected, was not regulated by Pos19 in neither wild-type nor Δ*hfq* background (Fig 5A). In addition to the microarray results, data from a target prediction by IntaRNA analysis [29,30] pointed to the possibility of RSP_0557 and *cysH* to be targets of Pos19 with interactions predicted for the translation initiation region, which we, therefore, tested in the reporter system. RSP_0557 expression was decreased to 32–37% in all tested Pos19 over-expression constructs (Fig 5A). These data support the observations from qRT-PCR experiments, where diminishment of mRNA levels was independent of an intact sORF. When the plasmid pPos19 was transferred to an *hfq* deletion mutant (Δ*hfq*), the negative effect on the *0557-lacZ* reporter was not observable (Fig 5A). Former results suggested that Pos19 is not an Hfq-binder [25] and the abolished function of Pos19 in the Δ*hfq* background was therefore surprising. Assuming that Pos19 might weakly bind to Hfq and has not overcome the detection limit in the former study, co-immunoprecipitation (coIP) with 3xFLAG-tagged Hfq was repeated with a strain carrying pPos19, demonstrating that Pos19 clearly binds Hfq when present in elevated amounts (Fig 5B). Since the *0557-lacZ* reporter carries parts of the RSP_0557 upstream region containing a potential promoter, an effect of Pos19 on the transcription of RSP_0557 could not be ruled out. The upstream region of RSP_0557 containing the potential promoter was transcriptionally fused to the *lacZ* gene containing its own ribosomal binding site. The activity of the RSP_0557 promoter did not differ in the empty vector control and Pos19 over-expression strain (S4 Fig).

To further evaluate the effect of Pos19 on *cysH*, we tested *cysH* in the *in vivo* reporter system together with two photosynthesis genes (*bchN* and *puc2A*), which were not affected in the Pos19 microarray. All three *lacZ*-fusions showed reduction of activity to 69–73% upon Pos19 over-expression (S5 Fig). Since the observed regulation was not expected for *bchN* and *puc2A*, the corresponding fusions were additionally tested in a Δ*hfq* background, where regulation was partly abolished (S5 Fig).

## Discussion

*Rhodobacter* is an established model organism for the investigation of photo-oxidative stress caused by ^1^O_2_ generated in photosynthetic complexes [1]. There is growing evidence that the major regulator of the photo-oxidative stress response in *R*. *sphaeroides*, RpoE, is activated by both ^1^O_2_ and diverse peroxides, including H_2_O_2_ and tBOOH [26,7], with similar observation made for *Caulobacter crescentus* [31]. The expression pattern of the here presented sRNA Pos19 supports this view (Fig 1B). It is known that ^1^O_2_ generates lipid and protein peroxides as secondary damages [13] and it is, therefore, possible that part of the RpoE regulon has a function in defending peroxide stress. The presented data provides evidence for a connection between oxidative stress and modulation of glutathione metabolism. Intriguingly, this connection is realized by an sRNA that was formerly found to be induced by ^1^O_2_ and which we renamed here as Pos19. Although the exact mechanism needs to be unraveled, Pos19 uniformly represses a set of 14 genes with a direct or indirect function in sulfur metabolism (Fig 3B and S2 Fig). A drop in sulfur availability will also restrict the biosynthesis of the sulfur-containing amino acid cysteine. Moreover, two of the 14 genes directly affect cysteine metabolism *(cysI* and *cysK*; S2 Fig). This leads to the assumption that the reduction of GSH, as seen for the Pos19 over-expression (Fig 4A), might be due to a reduction in cysteine, which is a major determinant for GSH production [32]. It is, therefore, more than surprising that Pos19 is induced as part of the RpoE regulon and in turn represses sulfur genes, thereby decreasing GSH levels. It seems intuitive that sulfur and cysteine metabolism should be triggered under oxidative stress conditions in order to provide maximal levels of GSH because GSH together with glutaredoxin (Grx) has a major contribution to oxidative stress resistance in *Rhodobacter* [33]. In fact, our data show that sulfur genes are clearly induced by ^1^O_2_ when Pos19 is not over-expressed (Fig 3). We, therefore, conclude that Pos19 slightly counterbalances this induction and thereby avoids overshooting sulfur levels in the cell. There are different aspects why this fine-tuning of sulfur genes and consequently the cysteine metabolism could have physiological benefits for the cell. Despite being used for GSH synthesis, cysteines can function as efficient reductants of Fe^3+^ and thereby drive the generation of hydroxyl radicals in the Fenton reaction [34]. Moreover, cysteines are directly damaged by ^1^O_2_ to form disulfides [13]. The key to understanding the balancing effect of Pos19 on sulfur gene expression is rather the risk of having too much cysteine than not having enough GSH. Indeed physiological assays, such as the zone of inhibition and ROS measurements, revealed that in the Pos19 over-expression strain the resistance towards oxidative stress was not affected in comparison to a control strain, even though GSH levels were decreased (Fig 4). A more general explanation why Pos19 would counteract expression of genes under oxidative stress addresses cellular resources. Stress defense systems have to be carefully regulated in order to avoid their unnecessarily high expression [35]. This ensures that resources that could be utilized for growth are not wasted [36]. Surprisingly, even though sulfur genes are up-regulated in the wild-type under ^1^O_2_ stress, they do not exhibit a stronger induction in the Pos19 deletion mutant. Additionally, a constitutive over-expression of Pos19 under non-stress conditions does not lead to reduced mRNA levels of the sulfur genes (data not shown). This leads to the assumption that there are redundant systems, which compensate for both constitutive presence and absence of Pos19. The observation that Pos19 mutants have altered GSH levels (Fig 4A) is conflicting and cannot be explained by regulation of sulfur genes. Maybe GSH biosynthesis is affected otherwise. One possible source for an altered GSH level in the Pos19 mutants might be regulation of the *cox* operon, encoding a putative carbon monoxide dehydrogenase. The *cox* operon was shown to be differentially regulated in our Pos19 microarray (Table 1). From a study investigating the CcsR1-4 sRNAs, it is known that the *cox* genes are part of a pathway that influences GSH levels [18]. A possible reason for an elevated GSH level in the Pos19 deletion mutant is an increased level of ROS, which triggers defense systems including the GSH biosynthesis. And indeed, a clear increase in ROS levels in the Pos19 deletion mutant was observed (Fig 4C). Even though the Pos19 deletion mutant did not show changed resistance towards oxidative stress, we observed an impaired growth under iron limitation, a condition that harbors increased ROS and induced Pos19 expression (S3 Fig). These data indicate that the role of Pos19 might be more complex and regulation of sulfur metabolism might only represent one aspect of this coding sRNA.

The function of the small peptide encoded by the sORF within the Pos19 sequence is still enigmatic. Our experiments however strongly indicate that regulation of several mRNAs does not depend on the sORF (Fig 3C). We considered *cysH* as a potential target of Pos19, but only found weak regulation in the *lacZ* reporter assay, which was comparable to regulation of the photosynthesis genes *bchN* and *puc2A* (S5 Fig). Even though not further examined here, Pos19 might be directly or indirectly involved in photosynthesis gene regulation. We focused on the hypothetical protein RSP_0557 as a Pos19 target and a potential missing link between Pos19 and the oxidative stress defense. RSP_0557 showed the highest expression change in our microarray data and is preceded by a putative RpoH_I_/H_II_-dependent promoter. Furthermore, RSP_0557 is homologous to RSP_6037 which is transcribed as well from a RpoH_I_/H_II_-dependent promoter [18] and is also down-regulated by Pos19 over-expression (Table 1). We could show a negative effect of Pos19 on RSP_0557 in various experiments, including a dependency of Pos19 on Hfq for regulation of RSP_0557 (Fig 5) and exclude regulation of RSP_0557 on transcriptional level (S4 Fig). Despite these findings, a direct interaction between Pos19 and the RSP_0557 mRNA could neither be shown via compensatory mutations nor with the help of electrophoretic mobility shift assays (data not shown). Unfortunately, we cannot ascribe a function to RSP_0557 so far. Protein BLAST searches identified the primosomal protein DnaI from *Roseobacter* to be 55% identical. Secondary structure predictions and comparisons by the Phyre^2^ program [37] revealed that RSP_0557 shows similarity to the RNA-binding protein Smaug with a confidence of 54.6%. RSP_0557 might have a regulatory function by binding to DNA or RNA. We hypothesized that RSP_0557 regulates sulfur genes and that the Pos19 effect on these genes is solely through RSP_0557. However, we could not validate this hypothesis in experiments with neither RSP_0557 deletion mutant nor over-expression strain (data not shown). Future studies are needed to elucidate the role of RSP_0557.

There are more intriguing questions. One addresses the small ORF within Pos19. Our *in vivo* data show that the ORF is translated and that mutations in the putative SD sequence do not completely abolish translation (Fig 2). This is in accordance with the growing evidence that the SD sequence in prokaryotes might not be as important for translation initiation as previously assumed [38,39]. Considering the Pos19 peptide has a function, Pos19 would represent another rare example of a dual-function sRNA. The most prominent members of this molecule class are RNAIII from *Staphylococcus aureus*, SgrS from *E*. *coli* and SR1 from *Bacillus subtilis* (for a review see [40]). In case of SgrS in *E*. *coli*, the small peptide SgrT inhibits the activity of the glucose-specific PTS transporter and therefore functions in the same regulatory pathway as the base-pairing sRNA SgrS [41]. A function of the Pos19 peptide in regulating the activity of sulfate or cysteine transporters is attractive but remains speculative.

In summary, our study identified Pos19 to be an sRNA regulator of sulfur genes that contributes to GSH homeostasis upon oxidative stress in *R*. *sphaeroides*. The remodeling of metabolic processes upon environmental changes is a mode of action becoming more and more prevalent for studied sRNAs (for review see [42]). Pos19 depicts, besides SorY [17], CcsR1-4 [18], and SorX [19] the fourth sRNA from *R*. *sphaeroides* alerting metabolic processes while adapting to stress conditions.

## Experimental Procedures

### Construction of a Pos19 deletion mutant

Chromosomal deletion of the *pos19* gene in *R*. *sphaeroides* 2.4.1 was achieved by homologous recombination. A 464 bp upstream and a 315 bp downstream fragment of the Pos19 locus were amplified using primer pairs 0019-up-Eco/ 0019-up-Pst and 0019-down-Sph/ 0019-down-Pst, respectively. The corresponding fragments were cloned into the EcoRI/SphI site of suicide plasmid pPHU281 [43] after digestion with EcoRI/PstI (upstream fragment) or SphI/PstI (downstream fragment). The kanamycin resistance cassette from plasmid pUC4K [44] was cloned into the newly generated PstI site between the upstream and downstream fragment. The resulting plasmid pPHU0019::Km was transferred to *R*. *sphaeroides* by conjugation and recombinants were selected on malate minimal salt medium agar plates containing kanamycin (25 μg ml^-1^). Recombinants were screened for tetracycline sensitivity to exclude single crossover events.

### Plasmid construction

All primers for plasmid construction are listed in Table A in S1 File and plasmids can be found in Table B in S1 File. For over-expression of Pos19, the *pos19* gene together with its native RpoE-dependent promoter was amplified with primers RSs0019_up and RSs0019_down using chromosomal *R*. *sphaeroides* DNA as template. The PCR product was sub-cloned into the pDrive cloning vector (Qiagen) and ligated into pBBR1 broad-host-range cloning vectors after digestion with suitable restriction enzymes. BamHI/HindIII and XbaI/PstI sites were used for pBBR1MCS-2 and pBBR1MCS-3, respectively. For site-directed mutagenesis, the pDrive derivative containing the Pos19 sequence was used as template for inverse PCR with primers containing the respective mutation (see Table A S1 File). Reaction mixtures were DpnI digested to deplete template plasmids. The correct PCR products were gel-purified using the QIAquick Gel Extraction Kit (Qiagen), transformed to *E*. *coli* JM109 by electroporation and screened by sequencing. Mutated Pos19 sequences were cloned into pBBR1 vectors as described above and resulting over-expression plasmids transferred to *R*. *sphaeroides* by biparental conjugation using *E*. *coli* S17-1.

For constitutive over-expression of Pos19 from a 16S rRNA promoter, the *pos19* gene was amplified with primers RSs0019_for_Bam and RSs0019_rev_Eco and inserted into the BamHI/EcoRI site of expression vector pRK4352 [45]. The resulting plasmid (pRK16S::Pos19) was transferred to wild-type 2.4.1 by conjugation.

For construction of fusion plasmids reporting on Pos19-sORF translation, the Pos19-sORF was amplified together with its native RpoE-dependent promoter via PCR using primers RSs0019_up and 0019-ORF-Hind. The PCR product was sub-cloned into the pDrive cloning vector (Qiagen) and ligated into pPHU236 after digestion with EcoRI and HindIII, resulting in a translational fusion of the Pos19-sORF and the promoter-less *lacZ* gene on pPHU236. Mutations in the Pos19 sequence were introduced by inverse PCR of the pDrive intermediate with primers containing the respective mutation (see Table A in S1 File). The Pos19-sORF fusion to eCFP (Pos19^up+ORF^::eCFP) was constructed using primers 0019_ORF_fw and 0019_ORF_rev, amplifying the Pos19 sORF without stop codon and a 154 bp upstream region. After sub-cloning into pDrive, the fragment was ligated into the corresponding sites of pBE4352::eCFP::eCFP [46] after digestion with XbaI and EcoRI. The resulting plasmid expresses the Pos19 peptide, C-terminally tagged with eCFP, from its own promoter. The plasmids were transferred to strains ΔPos19 and TF18 by conjugation.

For *in vivo* reporter studies of putative Pos19 targets, we made use of the pPHU4352 expression plasmid [45]. A 320 bp upstream fragment of RSP_0557 containing the first 10 codons was amplified with primers up-0557-for-Xho and up-0557-rev-Hind and cloned into the XhoI/HindIII sites of pPHU4352. For the *cysH* reporter, a 270 bp upstream fragment containing the first 10 codons was amplified with primers up-cysH-for-Bam and up-cysH-rev-Hind. The fragment was digested with BamHI/HindIII and cloned into the corresponding sites of pPHU4352. The RSP_0557 promoter fusion (P_0557_-*lacZ*) was constructed by amplifying a 149 bp upstream fragment of RSP_0557 using primers GA_P0557_fw and GA_P0557_rev. The PCR product was sub-cloned into pDrive cloning vector (Qiagen) and ligated into pBBR1MCS-3-*lacZ* [47] after digestion with AgeI and XbaI. The resulting reporter plasmids were transferred to *R*. *sphaeroides* strains by conjugation.

### Bacterial growth and stress experiments

*R*. *sphaeroides* strains (Table C in S1 File) were cultivated in malate minimal salt medium [14] at 32°C in the dark. Conditions of semi-aerobic (~25 μM dissolved oxygen) and aerobic growth (~180 μM dissolved oxygen) were accomplished as recently described [28]. Iron-limiting conditions were achieved as described in [15]. Stress experiments were conducted in exponential growth phase (OD_660_ of 0.4). Singlet oxygen (^1^O_2_) was generated by the addition of 0.2 μM methylene blue (Sigma-Aldrich) in the presence of high light intensities (800 W m^-2^ white light). Hydrogen peroxide (H_2_O_2_; Roth) and *tert*-butyl hydroperoxide (tBOOH; Sigma-Aldrich) were added in final concentrations of 1 mM and 300 μM, respectively. 250 μM of paraquat (PQ) were used for the generation of superoxide radicals (O_2_^‒^). Samples taken at the indicated time-points were rapidly cooled on ice and harvested by centrifugation at 10,000 x g in a chilled centrifuge.

### Measurement of sensitivity to ROS

Sensitivity to ROS was measured by inhibition zone assays. *R*. *sphaeroides* cultures were grown under semi-aerobic conditions until an OD_660_ of 0.4 was reached. A volume of 0.2 ml culture was mixed with 5 ml of pre-warmed top agar (0.8% agar) and layered on 15 ml malate minimal salt medium agar plates (1.6% agar). Filter paper disks were soaked with 5 μl solution of stress-inducing chemicals [10 mM methylene blue (Sigma-Aldrich), 0.5 M tBOOH (Sigma-Aldrich), 1 M and 2 M H_2_O_2_ (Roth), 0.5 M Diamide (Sigma-Aldrich)] and placed on the surface of the dried top agar. Zones of inhibition were measured after incubation at 32°C for 48 hours. Methylene blue plates were incubated under a fluorescent tube (model NL 36 W/860 daylight; Spectralux Plus, Radium), while tBOOH, H_2_O_2_, and diamide plates were incubated in the dark.

### Beta-galactosidase activity assay

Beta-galactosidase activities were measured in cell extracts from *R*. *sphaeroides* cultures. Measurements were carried out as described previously [43].

### Determination of total glutathione

For glutathione (GSH) assays, cultures were grown under semi-aerobic conditions to an OD_660_ of 0.4. Cells from 2 ml culture were harvested on ice and pellets were stored at -20°C after centrifugation at 10,000 x g. Sample preparation and GSH measurements were carried out as recently described using Ellman’s reagent [18].

### Measurement of ROS level

Reactive oxygen species (ROS) generation was measured using the oxidation-sensitive fluorescent probe 2,7-dihydrodichlorofluorescein diacetate (DCFH-DA; Molecular Probes). Cells were incubated with the probe at a final concentration of 10 μM for 30 min. The fluorescence intensities (excitation 492 nm, emission 525 nm) were evaluated in an Infiniti M200 microplate reader (Tecan).

### Western blotting

For Western blot experiments, *R*. *sphaeroides* cultures were grown under semi-aerobic and ^1^O_2_ stress conditions as described above. Samples were taken at the indicated time points. Cells were broken by sonication and cell debris removed by centrifugation. Total protein extracts were separated on 10% PAA-SDS gels and transferred to nitrocellulose membranes (Whatman). Proteins were stained and fixed with Ponceau S (Sigma-Aldrich) and de-stained with sodium hydroxide. Membranes were blocked for 1 hour at room temperature with blocking buffer (1x TBS) containing 5% (w/v) milk powder (Roth). After blocking, the purified primary antibody α-GFP, diluted 1:4000 in blocking buffer, was added to the membrane and incubated for 1 hour. After washing the membrane 3 times for 5 min in 1x TBS buffer, the secondary antibody (anti-mouse IgG conjugated with horseradish peroxidase, produced in goat, Sigma-Aldrich) was added (diluted 1:5000 in blocking buffer) and the membrane further incubated for 1 hour at room temperature. The membrane was washed 3 times with 1x TBS for 5 minutes. Western blots were developed using the Lumi-Light Western Blotting Substrate 1 and 2 (Roche) and analyzed on a Chemiluminescence-Imaging System (Fusion-SL4; Peqlab).

### Northern blotting

Total RNA was prepared using three hot phenol steps [48]. Five μg RNA were separated on 10% polyacrylamide gels containing 7 M urea. RNA was transferred to Roti-Nylon plus 0.45 μm membranes (Roth) by semi-dry electroblotting. For detection, oligodeoxynucleotides (Table A in S1 File) were labeled with [γ-^32^]-ATP (Hartmann Analytik) as described in [16]. Pre-hybridization and hybridization were performed in low-stringency buffer [49] at 42°C. Membranes were washed with solutions containing 5x SSC and 0.01% SDS. Membranes were exposed to phosphorimaging screens (Bio-Rad) and analyzed with the 1D-Quantity One software (Bio-Rad).

### Microarray analysis

For gene expression studies, RNA was isolated and hybridized to Custom Gene Expression Microarrays from Agilent Technologies (8x15K; ID: 027061) as recently described [28]. The ULS Fluorescent Labeling Kit for Agilent arrays (Kreatech) was used for Cy3/Cy5 labeling (two-color microarrays) and subsequent steps were performed with reagents from Agilent Technologies. Scanning was performed with the Agilent DNA microarray scanner and raw median fluorescence values were calculated using the Feature Extraction Software (Agilent). Within-array normalization according to LOESS was accomplished with the Bioconductor package Limma for R [50]. The data discussed in this publication have been deposited in NCBI’s Gene Expression Omnibus [51] and are accessible through GEO Series accession number GSE81718.

### Real-time RT-PCR

Total RNA for RT-PCR was isolated using the peqGOLD TriFast reagent (PeqLab) as described by the manufacturer. Contaminating DNA was removed by DNaseI (Invitrogen) treatment. Purification was performed by standard procedures, using a mixture of phenol-chloroform-isoamyl alcohol and chloroform-isoamyl alcohol. Real-time RT-PCR reactions were prepared using the Brilliant III Ultra-Fast SYBR Green QRT-PCR Master Mix (Agilent) with total RNA in a final concentration of 4 ng μl^−1^. Amplification was performed in a Corbett Research Rotor-Gene RG-3000 Thermal Cycler and analyzed with the Rotor-Gene Manager program. Relative mRNA levels were calculated according to Pfaffl [52]. The *rpoZ* gene was used as an endogenous control. Primers and their amplification efficiencies are listed in Tables A and D in S1 File, respectively.

### 3’ RACE

For detection of sRNA 3’ ends, 3’ RACE was performed as recently described [45]. In short, RNA from stress samples was applied to 3’-poly(A) tailing, using poly(A) polymerase (Ambion). Purified poly(A)-tailed RNA was used as template for RT-PCR with the OneStep RT-PCR Kit (Qiagen). Primers were oligo-dT and 0019–3’RACE-2 (Table A in S1 File). RT-PCR products were cloned into the pDrive cloning vector (Qiagen) for sequencing.

### Co-immunoprecipitation

*R*. *sphaeroides* strain 2.4.1-3xFLAG*hfq*^+^ was used for coIP of RNA with the Hfq protein as described earlier 25]. Control plasmid pBBR1 and over-expression plasmid pPos19 were transferred to 2.4.1-3xFLAG*hfq*^+^ as well as 2.4.1*hfq*^+^ (negative control). Resulting strains were exposed to ^1^O_2_ for 30 min at an OD_660_ of 0.4. Cell pellets from 150 ml culture (OD 60) were lysed by sonification and equivalent amounts of OD 6 and OD 54 were applied to total RNA (input) and coIP RNA (output) extraction, respectively. ANTI-FLAG M2 Affinity Agarose Gel (Sigma-Aldrich) was used for coIP of crude extracts. Six μg of total RNA and 350 ng of coIP RNA were loaded on polyacrylamide-gels for detection of Pos19 by Northern blot analysis.

## Supporting Information

S1 Fig Alignment of the Pos19 locus from four different *R*. *sphaeroides* strains. Strain 2.4.1 represents the wild-type strain used in this study. The promotor motif is indicated by -35 and -10, while +1 indicates the transcriptional start site. Start (ATG) and stop (TGA) codons of the sORF are marked in grey.
(TIF)

S2 Fig Sulfate metabolism and assimilatory sulfate reduction as illustrated in KEGG pathway maps (http://www.genome.jp/kegg/). *R*. *sphaeroides* genes (RSP), that have a function in these pathways and were shown to be affected by Pos19 in this study, are highlighted in red.
(TIF)

S3 Fig Pos19 expression and effect under iron limitation. (A) Northern blot of samples from *R*. *sphaeroides* wild-type grown in minimal malate medium (+Fe) and iron-limited medium (-Fe; for details see Remes *et al*., 2014). *The 5.8S rRNA signal stems from hybridization with probe p-0680a which is known to cross-hybridize to the 5.8S rRNA. (B) Growth curve of *R*. *sphaeroides* wildtype (black lines) and Pos19 mutant (grey lines) as biological triplicates each, in minimal malate medium (solid lines) and in iron-limited medium (dashed lines).
(TIF)

S4 Fig ß-galactosidase activity assay for the RSP_0557 promotor fusion. The plasmid P_0557_-*lacZ* carrying the RSP_0557 upstream region (-149 bp from TSS) in the empty vector control strain (pBBR) and the Pos19 over-expression strain (pPos19). Samples were taken after 60 min of ^1^O_2_ stress. The results represent the mean of technical duplicates from biological triplicates and the standard deviation from the mean.
(TIF)

S5 Fig ß-galactosidase activity assay for *takP*, *cysH*, *bchN*, and *puc2A* fusions. Gene fragments containing the first codons and part of the upstream region were translationally fused to the *lacZ* gene on plasmid pPHU235. The reporter plasmids were transferred to control (pBBR1) and over-expression strains of Pos19 (pPos19) in wild-type (wt 2.4.1) and *hfq* deletion mutant (Δ*hfq*) backgrounds. Cultures were stressed with ^1^O_2_ for 60 min and samples subjected to β-galactosidase assays. Bars indicate the relative β-galactosidase activity as calculated from Miller units (with the pBBR1 control set to 100% for each measurement). Results represent the mean from three independent experiments with technical duplicates and error bars reflect the standard deviation.
(TIF)

S1 File Oligodeoxynucleotides (Table A), plasmids (Table B), and strains (Table C) used in this study. As well as efficiencies for qRT-PCR primers (Table D).
(DOCX)

S1 Table Genes which are down-regulated upon Pos19 over-expression with a log2 ratio ≤ -0.6. (DOCX)
