# Supplementary figures and images for: Characteristics of Pos19 – A Small Coding RNA in the Oxidative Stress Response of Rhodobacter sphaeroides
Source: PLoS One. 2016 Sep 26;11(9):e0163425. doi: 10.1371/journal.pone.0163425 (PMC5036791; doi:10.1371/journal.pone.0163425)

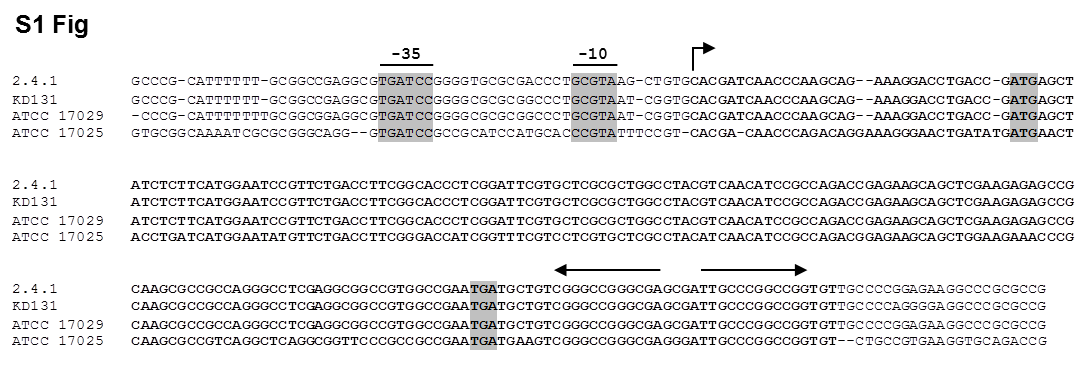

Supplement: S1 Fig — Strain 2.4.1 represents the wild-type strain used in this study. The promotor motif is indicated by -35 and -10, while +1 indicates the transcriptional start site. Start (ATG) and stop (TGA) codons of the sORF are marked in grey. (TIF) [file pone.0163425.s001.tif]

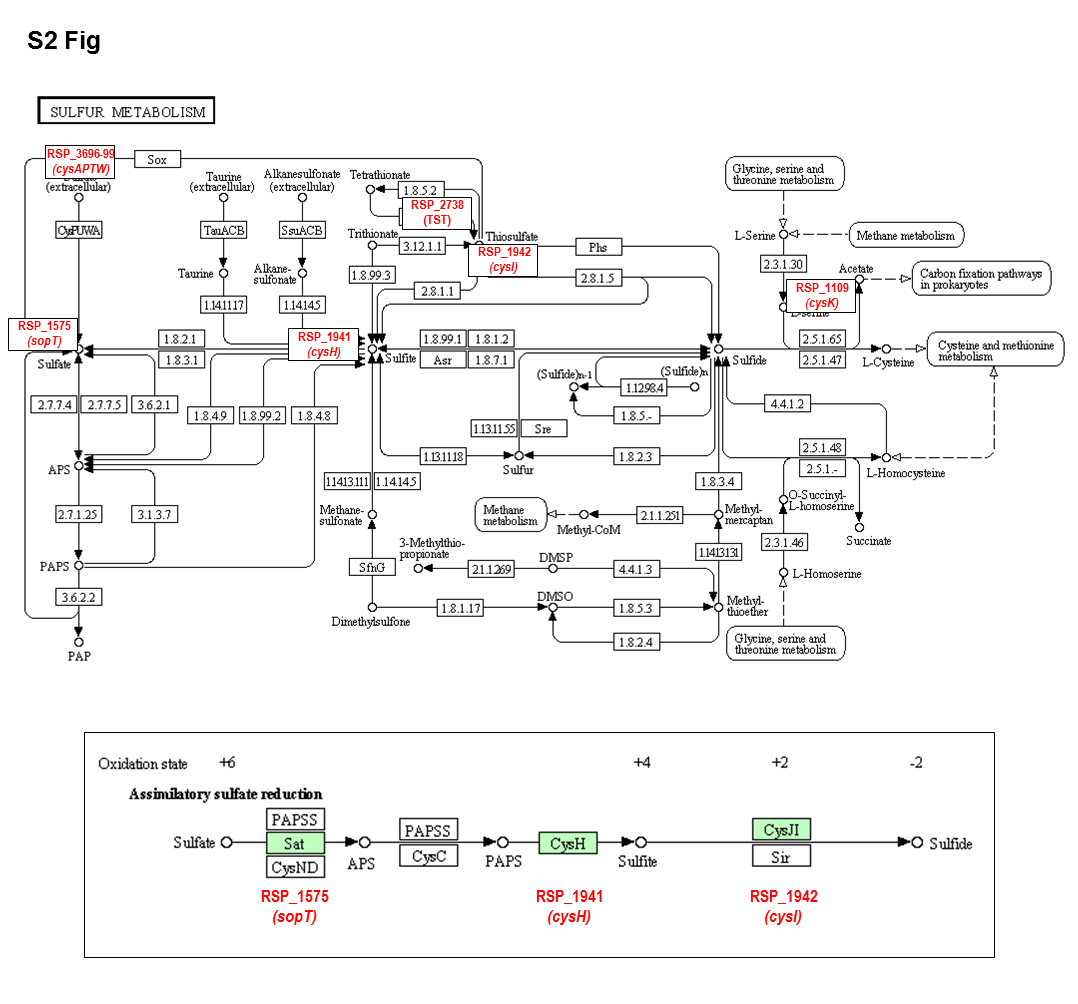

Supplement: S2 Fig — R. sphaeroides genes (RSP), that have a function in these pathways and were shown to be affected by Pos19 in this study, are highlighted in red. (TIF) [file pone.0163425.s002.tif]

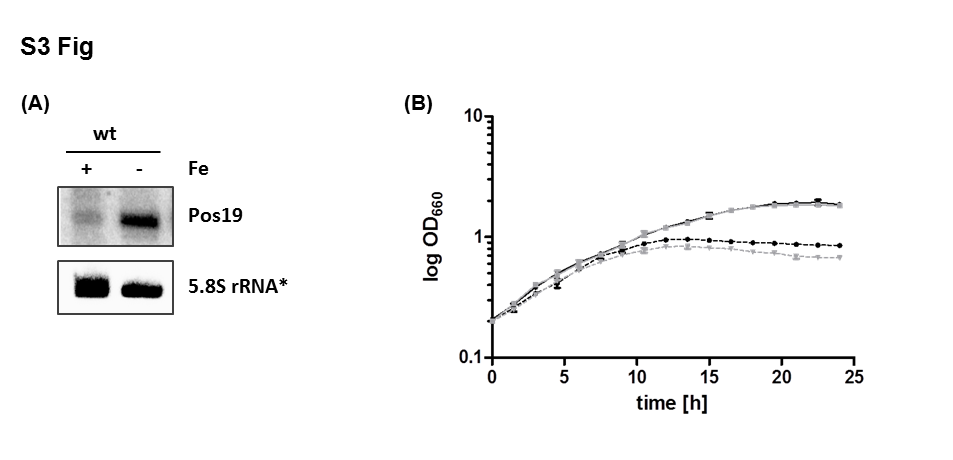

Supplement: S3 Fig — (A) Northern blot of samples from R. sphaeroides wild-type grown in minimal malate medium (+Fe) and iron-limited medium (-Fe; for details see Remes et al., 2014). *The 5.8S rRNA signal stems from hybridization with probe p-0680a which is known to cross-hybridize to the 5.8S rRNA. (B) Growth curve of R. sphaeroides wildtype (black lines) and Pos19 mutant (grey lines) as biological triplicates each, in minimal malate medium (solid lines) and in iron-limited medium (dashed lines). (TIF) [file pone.0163425.s003.tif]

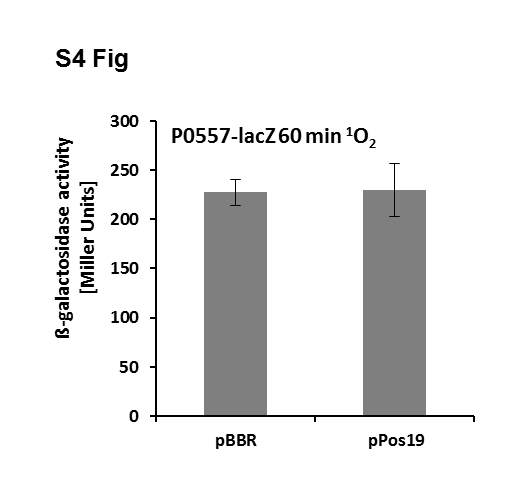

Supplement: S4 Fig — The plasmid P0557-lacZ carrying the RSP_0557 upstream region (-149 bp from TSS) in the empty vector control strain (pBBR) and the Pos19 over-expression strain (pPos19). Samples were taken after 60 min of 1O2 stress. The results represent the mean of technical duplicates from biological triplicates and the standard deviation from the mean. (TIF) [file pone.0163425.s004.tif]

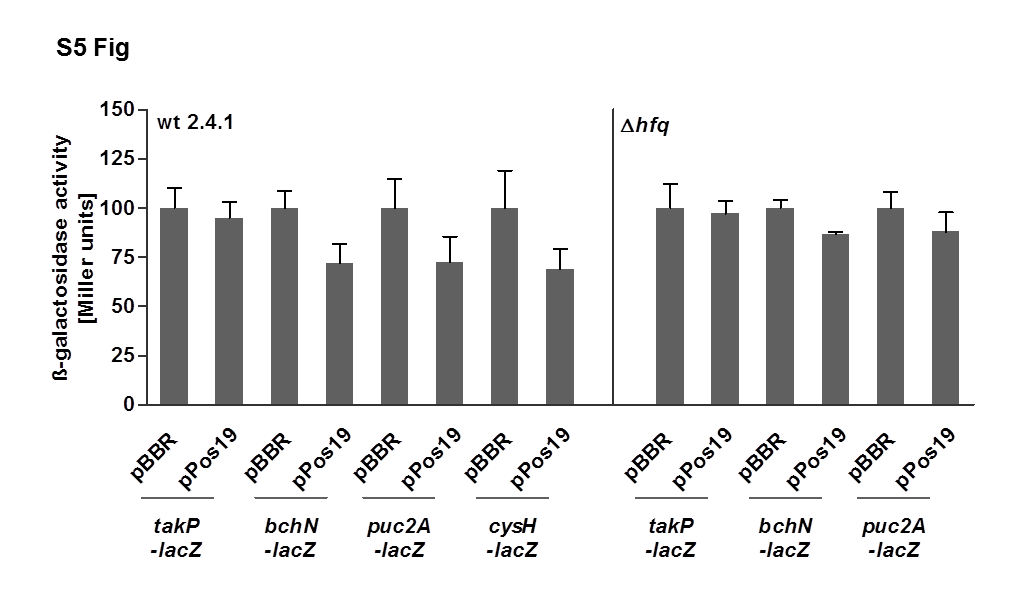

Supplement: S5 Fig — Gene fragments containing the first codons and part of the upstream region were translationally fused to the lacZ gene on plasmid pPHU235. The reporter plasmids were transferred to control (pBBR1) and over-expression strains of Pos19 (pPos19) in wild-type (wt 2.4.1) and hfq deletion mutant (Δhfq) backgrounds. Cultures were stressed with 1O2 for 60 min and samples subjected to β-galactosidase assays. Bars indicate the relative β-galactosidase activity as calculated from Miller units (with the pBBR1 control set to 100% for each measurement). Results represent the mean from three independent experiments with technical duplicates and error bars reflect the standard deviation. (TIF) [file pone.0163425.s005.tif]
