# Supplementary material for: Characteristics of Pos19 – A Small Coding RNA in the Oxidative Stress Response of Rhodobacter sphaeroides
Source: PLoS One. 2016 Sep 26;11(9):e0163425. doi: 10.1371/journal.pone.0163425 (PMC5036791; doi:10.1371/journal.pone.0163425)
Supplement: S1 File — Oligodeoxynucleotides (Table A), plasmids (Table B), and strains (Table C) used in this study. As well as efficiencies for qRT-PCR primers (Table D). (DOCX) [file pone.0163425.s006.docx]

**S1 file Table A**

**Name Sequence (5’ to 3’) Purpose**

oligo-dT primer GACCACGCGTATCGAGTCGACTTTTTTTTTTTTTTTTV 3’ RACE

0019-3’RACE-2 CTACGTCAACATCCGCCAGACC 3’ RACE

p-0019 GAGATAGCTCATCGGTCAGGTCC Northern

p-5S CTTGAGACGCAGTACCATTG Northern

p-0680a CGTCGCCGCTGCTGCTACAGGTC Northern

0019-up-Eco GAATTCGCTCTACCGGCCGCACGAG Cloning

0019-up-Pst CTGCAGCCTGATCACGCCTCGGCCG Cloning

0019-down-Sph CTGCAGGAGCTTCCGACGACGCGAG Cloning

0019-down-Pst GCATGCGCGTGGCGGCCTTCCTCG Cloning

0557-up-EcoRI cgcGAATTCatattcgccgaccgccag Cloning

0557-up-PstI cgcCTGCAGgaagtggaaggtggagatgg Cloning

0557-down-SphI cgcGCATGCcctatctcgcccaaggattc Cloning

0557-down-Pst-2 cgcCTGCAGggcgatatcgatgacatctgc Cloning

RSs0019_up CGTGGTGGTCGGCTGAAC Cloning

RSs0019_down GGCGGCAGGAAGGATGTC Cloning

RSs0019_for_Bam GGATCCCGATCAACCCAAGCAGAA Cloning

RSs0019_rev_Eco GAATTCGGATGTCCCGCTCAGG Cloning

0019-ORF-Hind aagcttTCGGCCACGGCCGCCTCG Cloning

0019-ORF-fw TGGTCTAGACGTGGTGGTCGGCTGAACCACCCG Cloning

0019-ORF-rev TGGCATATGTTCGGCCACGGCCGCCTCGAGGCCCTG Cloning

up-0557-for-Xho CTCGAGgctagcacgaagcgggaacacctg Cloning

up-0557-rev-Hind AAGCTTcgccgggcgggtcgtctcgaag Cloning

up-cysH-for-Bam GGATCCgcgacggcaccgagacggcg Cloning

up-cysH-rev-Hind AAGCTTgtgcttgtagcgaccgttgagc Cloning

0019_Stop16_fw CACCCTCTGATTCGTGCTCGCGCTGGCC Cloning

0019_Stop16_rev CACGAATCAGAGGGTGCCGAAGGTCAG Cloning

0019_Stop1_fw CTGACCGTGAAGCTATCTCTTCATGGAATC Cloning

0019_Stop1_rev ATAGCTTCACGGTCAGGTCCTTTCTGCTTG Cloning

0019_Thr1_fw CTGACCGACGAGCTATCTCTTCATGGAATC Cloning

0019_Thr1_rev ATAGCTCGTCGGTCAGGTCCTTTCTGCTTG Cloning

0019_Stop2_fw CTCTTCTGAGAATCCGTTCTGACCTTCG Cloning

0019_Stop2_rev CGGATTCTCAGAAGAGATAGCTCATCGG Cloning

0019_Thr2_fw CTCTTCACGGAATCCGTTCTGACCTTCG Cloning

0019_Thr2_rev CGGATTCCGTGAAGAGATAGCTCATCGG Cloning

0019_SD-M4_fw CAAGCAGAATCCTCCTGACCGATGAGCTATC Cloning

0019_SD-M4_rev AGGAGGATTCTGCTTGGGTTGATCGTGCAC Cloning

0019_SD-M8_fw CAAGCTCTTTCCTCCTGACCGATGAGCTATC Cloning

0019_SD-M8_rev AGGAGGAAAGAGCTTGGGTTGATCGTGCAC Cloning

GA_P0557_fw GGTAGTCAATAAACCGGTCTTCTTCCGCGTGATAC Cloning

GA_P0557_rev GGGATCCACTAGTTCTAGAAACAGGCAAATAAGCC Cloning

1669-A ATCGCGGAAGAGACCCAGAG RT-PCR

1669-B CATCAGCTGGTAGCTCTC RT-PCR

0557-A CCATCTCCACCTTCCACTTC RT-PCR

0557-B CAGAGACCGATGTCGTCGAG RT-PCR

0130-A CACGCTTCAGACGCTCTACA RT-PCR

0130-B GAATAGCCGATGAGGCTGAC RT-PCR

1351-A CCTATTCGCTGGACCTTCTG RT-PCR

1351-B GTCGCAGATGGTGAGACCTT RT-PCR

1941-A TGCATCTCGTCTCGGTCATC RT-PCR

1941-B TCGGTGTTGTAGCGGTTCAG RT-PCR

1942-A AGTTCAAGATCGCCATGACC RT-PCR

1942-B GCCCGGTAGAAATCGTCATA RT-PCR

3697-A AATGCGCGCTACACCTATCT RT-PCR

3697-B GTCATGCACCCGGTAGAAAT RT-PCR

0799-A GAACAATTACGCCTTCTC RT-PCR

0799-B CATCAGCTGGTAGCTCTC RT-PCR

**S1 file Table B**

**Plasmid Description Source/reference**

pDrive cloning vector, Ap^r^, Km^r^ Qiagen

pUC4K source of Km^r^ cassette, Km^r^ Vieira & Messing (1982)

pPHU281 Suicide plasmid for *R. sphaeroides*, Tc^r^ Hübner *et al.* (1991)

pBBR1MCS-2 Broad-host-range cloning vector, pBBR1MCS derivative, Km^r^ Kovach *et al.* (1995)

pBBR1MCS-3 Broad-host-range cloning vector, pBBR1MCS derivative, Tc^r^ Kovach *et al.* (1995)

pBBR1MCS-3-*lacZ* Broad-host-range *lacZ* fusion vector, Tc^r^ Fried *et al*. (2012)

pPHU235 Broad-host-range *lacZ* fusion vector, Tc^r^ Hübner *et al.* (1991)

pPHU236 Broad-host-range *lacZ* fusion vector, Tc^r^ Hübner *et al*. (1991)

pPHU0019::Km pPHU281 derivative for *pos19* deletion, Tc^r^ This study

pPHU0557::Km pPHU281 derivative for RSP_0557 deletion, Tc^r^ This study

pPos19 pBBR1 containing Pos19 with its own promoter, Km^r^ or Tc^r^ This study

pPos19-Stop16 pPos19 with codon 16 GGA to TGA exchange, Km^r^ or Tc^r^ This study

pPos19-Stop1 pPos19 with start codon 1 ATG to TGA exchange, Km^r^ or Tc^r^ This study

pPos19-Thr1 pPos19 with start codon 1 ATG to ACG exchange, Km^r^ or Tc^r^ This study

pPHU-wtORF pPHU236 containing Pos19-ORF fused to *lacZ*, Tc^r^ This study

pPHU-Stop16 pPHU-wtORF with codon 16 GGA to TGA exchange, Tc^r^ This study

pPHU-Stop1 pPHU-wtORF with start codon 1 ATG to TGA exchange, Tc^r^ This study

pPHU-Thr1 pPHU-wtORF with start codon 1 ATG to ACG exchange, Tc^r^ This study

pPHU-Stop2 pPHU-wtORF with start codon 2 ATG to TGA exchange, Tc^r^ This study

pPHU-Thr2 pPHU-wtORF with start codon 2 ATG to ACG exchange, Tc^r^ This study

pPHU-M4 pPHU-wtORF with SD AGGA to TCCT exchange, Tc^r^ This study

pPHU-M8 pPHU-wtORF with SD AGAAAGGA to TCTTTCCT exchange, Tc^r^ This study

pBE4352::eCFP::eCFP *ecfp* fusion vector, Km^r^ Remes *et al.* (2015)

pBE::Pos19^up+ORF^::eCFP Pos19-sORF fusion to *ecfp* with own promoter, Km^r^ This study

pRK4352 pRK415 containing RSP_4352 promoter, Tc^r^ Mank *et al.* (2102)

pRK16S::Pos19 pRK4352 containing the *pos19* gene, Tc^r^ This study

pPHU4352 pPHU235 containing RSP_4352 promoter, Tc^r^ Mank *et al.* (2012)

pPHUpuc2A pPHU4352 containing *puc2A* fragment for *lacZ* fusion, Tc^r^ Mank *et al*. (2012)

pPHUbchN pPHU4352 containing *bchN* fragment for *lacZ* fusion, Tc^r^ Mank *et al*. (2012)

pPHU0557 pPHU4352 containing RSP_0557 fragment for *lacZ* fusion, Tc^r^ This study

pPHUcysH pPHU4352 containing *cysH* fragment for *lacZ* fusion, Tc^r^ This study

pBBR 0557up-*lacZ*  pBBR1 *lacZ* containing RSP_0557 fragment for *lacZ* fusion, Tc^r^ This study

**S1 file Table C**

**Strain Description Source/reference**

*E. coli*

S17-1 *tra*^+^ for diparental conjugation Simon *et al*. (1986)

JM109 Host strain for plasmid construction New England Biolabs

*R. sphaeroides*

2.4.1 Wild type van Niel (1944)

2.4.1*hfq*::ΩSp *hfq* mutation in 2.4.1 (Δ*hfq*), Sp^r^ Glaeser *et al*. (2007)

2.4.1*hfq*^+^ 2.4.1Δ*hfq* harbouring pRK2.4.1*hfq*, Sp^r^, Tc^r^ Glaeser *et al*. (2007)

2.4.1-3xFLAG*hfq*^+^ 2.4.1Δ*hfq* harbouring pRK2.4.1-3xFLAG*hfq*, Sp^r^, Tc^r^ Berghoff *et al*. (2011)

TF18 *rpoE chrR* mutation in 2.4.1, Tp^r^ Schilke & Donohue (1995)

Δ*rpoHI* *rpoHI* mutation in 2.4.1, Km^R^ Nuss *et al*. (2010)

Δ*rpoHII* *rpoHII* mutation in 2.4.1, Km^R^ Nuss *et al*. (2009)

2.4.1*pos19*::Km *pos19* mutation in 2.4.1 (ΔPos19), Km^r^ This study

**S1 file Table C**

**Gene Efficiency**

RSP_1669 (*rpoZ*) 2.02

RSP_0557 2.04

RSP_0130 (*metI*) 1.91

RSP_1351 (*serC*) 1.94

RSP_1941 (*cysH*) 1.76

RSP_1942 (*cysI*) 1.95

RSP_3697 (*cysP*) 2.14

RSP_0799 (*gloB*) 1.99

**Supp References**

Berghoff BA, Glaeser J, Sharma CM, Zobawa M, Lottspeich F, Vogel J, et al. Contribution of Hfq to photooxidative stress resistance and global regulation in *Rhodobacter sphaeroides*. Mol Microbiol. 2011 Jun;80(6):1479-95.

Fried L, Lassak J, Jung K. A comprehensive toolbox for the rapid construction of *lacZ* fusion reporters. J Microbiol Methods. 2012 Dec;91(3):537-43.

Glaeser J, Zobawa M, Lottspeich F, Klug G. Protein synthesis patterns reveal a complex regulatory response to singlet oxygen in *Rhodobacter*. J Proteome Res. 2007 Jul;6(7):2460-71.

Hübner P, Willison JC, Vignais PM, Bickle TA. Expression of regulatory nif genes in *Rhodobacter capsulatus*. J Bacteriol. 1991 May;173(9):2993-9.

Kovach ME, Elzer PH, Hill DS, Robertson GT, Farris MA, Roop RM, 2nd, et al. Four new derivatives of the broad-host-range cloning vector pBBR1MCS, carrying different antibiotic-resistance cassettes. Gene. 1995 Dec 1;166(1):175-6.

Mank NN, Berghoff BA, Hermanns YN, Klug G. Regulation of bacterial photosynthesis genes by the small noncoding RNA PcrZ. Proc Natl Acad Sci USA. 2012 Oct 2;109(40):16306-11.

Nuss AM, Glaeser J, Klug G. RpoH(II) activates oxidative-stress defense systems and is controlled by RpoE in the singlet oxygen-dependent response in *Rhodobacter sphaeroides*. J Bacteriol. 2009 Jan;191(1):220-30.

Nuss AM, Glaeser J, Berghoff BA, Klug G. Overlapping alternative sigma factor regulons in the response to singlet oxygen in *Rhodobacter sphaeroides*. J Bacteriol. 2010 May;192(10):2613-23.

Remes B, Eisenhardt BD, Srinivasan V, Klug G. IscR of *Rhodobacter sphaeroides* functions as repressor of genes for iron-sulfur metabolism and represents a new type of iron-sulfur-binding protein. Microbiologyopen. 2015 Oct;4(5):790-802.

Schilke BA, Donohue TJ. ChrR positively regulates transcription of the *Rhodobacter sphaeroides* cytochrome c2 gene. J Bacteriol. 1995 Apr;177(8):1929-37.

Simon R, O'Connell M, Labes M, Puhler A. Plasmid vectors for the genetic analysis and manipulation of rhizobia and other gram-negative bacteria. Methods Enzymol. 1986;118:640-59.

van Niel CB. The culture, general physiology, morphology, and classification of the non-sulfur purple and brown bacteria. Bacteriol Rev. 1944;8:1-118.

Vieira J, Messing J. The pUC plasmids, an M13mp7-derived system for insertion mutagenesis and sequencing with synthetic universal primers. Gene. 1982 Oct;19(3):259-68.
