# Supplementary material for: Characteristics of Pos19 – A Small Coding RNA in the Oxidative Stress Response of Rhodobacter sphaeroides
Source: PLoS One. 2016 Sep 26;11(9):e0163425. doi: 10.1371/journal.pone.0163425 (PMC5036791; doi:10.1371/journal.pone.0163425)
Supplement: S1 Table — (DOCX) [file pone.0163425.s007.docx]

**S1 Table**

**Log_2_ ratio**

**Gene Number Gene Name pPos19 vs. pBBR1 Function**

**With function in sulfur metabolism**

RSP_0129 *metN* -0.80 ABC D-methionine uptake transporter, ATPase subunit

RSP_0130 *metI* -0.80 ABC D-methionine uptake transporter, inner membrane subunit

RSP_0132 *metQ* -0.68 ABC D-methionine uptake transporter, substrate-binding protein

RSP_0437 *sufC* -0.61

RSP_1109 *cysK* -0.67 Cysteine synthase

RSP_1351 *serC* -0.98 Phosphoserine aminotransferase

RSP_1352 *serA* -1.03 D-3-phosphoglycerate dehydrogenase

RSP_1575 sopT -0.82 Sulfate adenylyltransferase

RSP_1941 *cysH* -1.06 Phosphoadenosine phosphosulfate reductase

RSP_1942 *cysI* -1.25 Sulfite/nitrite reductase hemoprotein subunit

RSP_1943 -1.39 Sulfite reductase (ferredoxin)

RSP_1944 *cysG/cobA* -1.29 Uroporphiryn-III C-methyltransferase/siroheme synthase

RSP_2738 TST -0.79 Probable Rhodanese-related sulfurtransferase

RSP_3697 *cysP* -0.74 ABC sulfate/thiosulfate transporter, periplasmic binding protein

**Without function in sulfur metabolism**

RSP_0147 *glnA*  -0,62 Glutamine synthetase class-I

RSP_0557 -2.30 Hypothetical protein

RSP_1110 -0.65 Hypothetical protein

RSP_1547 -0.69 Probable bacterioferritin-associated ferredoxin

RSP_1987 -0.59 Hypothetical protein

RSP_2017 -0.72 Hypothetical protein

RSP_2115 *envA* -0.70 Putative UDP-3-O-acyl N-acetylglucosamine deacetylase

RSP_2739 -0.63 Hypothetical protein

RSP_3095 -0.62 RNA polymerase sigma-70 factor

RSP_3715 -0.66 pH adaption potassium efflux system, PhaB subunit

RSP_4254 *rnk* -0.92 putative regulator of nucleoside diphosphate kinase protein

RSP_4255 -0.89 Mechanosensitive (MS) ion channel protein

RSP_6029 -0.61 Hypothetical protein

RSP_6037 -1.04 Hypothetical protein

RSP_6066 -0.60 Hypothetical protein

RSP_6180 -0.61 Hypothetical protein

Log_2_ ratios were calculated from two individual microarray analyses, each containing pooled biological triplicates per strain. Reproducibility was high as reflected by a correlation coefficient (Pearson) of *r* = 0.92. Genes located in an operon are grouped together.
